# *N*-(2,4-Dinitro­phen­yl)-1,3-dimeth­oxy­isoindolin-2-amine

**DOI:** 10.1107/S1600536811026316

**Published:** 2011-07-09

**Authors:** Na-Na Du, Hua-Jie Xu, Liang-Quan Sheng

**Affiliations:** a Department of Chemistry, Fuyang Normal College, Fuyang, Anhui 236041, People’s Republic of China

## Abstract

In the title compound, C_16_H_16_N_4_O_6_, the planes of the isoindole and dinitro­benzene groups make a dihedral angle between  of 84.15 (8)°. The N atom of the isoindole group is displaced by 0.2937 (3) Å from the plane through the remaining atoms. An intra­molecular N—H⋯O inter­action occurs. In the crystal, inversion dimers linked by pairs of N—H⋯O hydrogen bonds occur.

## Related literature

For general background to isoindoles and their derivatives, see: Mancilla *et al.* (2007 ▶); Toru *et al.* (1986 ▶). For the synthetic method and related structures, see: Maliha *et al.* (2008 ▶, 2009 ▶).
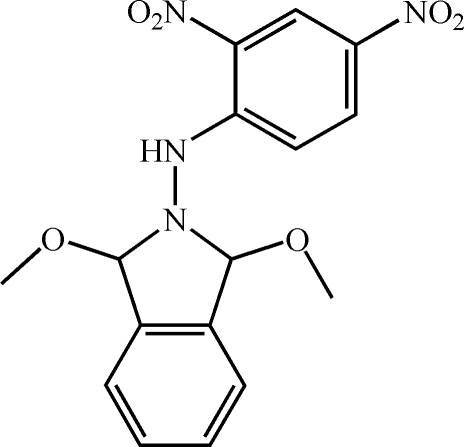


## Experimental

### 

#### Crystal data

C_16_H_16_N_4_O_6_
*M* *_r_* = 360.33
Triclinic, 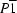

*a* = 7.727 (4) Å
*b* = 10.244 (5) Å
*c* = 11.326 (6) Å
α = 86.076 (9)°
β = 77.705 (8)°
γ = 70.794 (8)°
*V* = 827.2 (7) Å^3^
*Z* = 2
Mo *K*α radiation
μ = 0.11 mm^−1^
*T* = 296 K
0.16 × 0.14 × 0.10 mm

#### Data collection

Bruker SMART APEXII CCD diffractometer
Absorption correction: multi-scan (*SADABS*; Sheldrick, 1996 ▶) *T*_min_ = 0.982, *T*_max_ = 0.989
4365 measured reflections
3181 independent reflections
2045 reflections with *I* > 2σ(*I*)
*R*_int_ = 0.062

#### Refinement

*R*[*F* ^2^ > 2σ(*F* ^2^)] = 0.067
*wR*(*F* ^2^) = 0.225
*S* = 1.02
3181 reflections
237 parameters
H-atom parameters constrained
Δρ_max_ = 0.49 e Å^−3^
Δρ_min_ = −0.25 e Å^−3^

### 

Data collection: *APEX2* (Bruker, 2003 ▶); cell refinement: *SAINT* (Bruker, 2003 ▶); data reduction: *SAINT*; program(s) used to solve structure: *SHELXS97* (Sheldrick, 2008 ▶); program(s) used to refine structure: *SHELXL97* (Sheldrick, 2008 ▶); molecular graphics: *SHELXTL* (Sheldrick, 2008 ▶); software used to prepare material for publication: *SHELXTL*.

## Supplementary Material

Crystal structure: contains datablock(s) global, I. DOI: 10.1107/S1600536811026316/ez2248sup1.cif

Structure factors: contains datablock(s) I. DOI: 10.1107/S1600536811026316/ez2248Isup2.hkl

Supplementary material file. DOI: 10.1107/S1600536811026316/ez2248Isup3.cml

Additional supplementary materials:  crystallographic information; 3D view; checkCIF report

## Figures and Tables

**Table 1:** Hydrogen-bond geometry (Å, °)

*D*—H⋯*A*	*D*—H	H⋯*A*	*D*⋯*A*	*D*—H⋯*A*
N2—H2*A*⋯O1	0.86	1.96	2.593 (3)	130
N2—H2*A*⋯O1^i^	0.86	2.27	3.032 (3)	148

Symmetry code: (i) 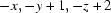.

**Fig. 1.**
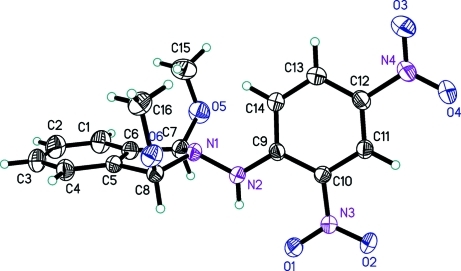
The molecular structure of the title molecule, with the atom-numbering scheme. Displacement ellipsoids are drawn at the 30% probability level.

**Fig. 2.**
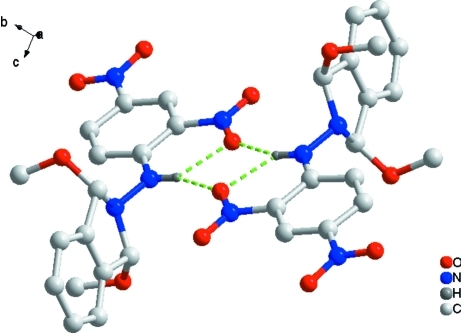
The dimeric structure of the title compound formed by intermolecular hydrogen bonds. The intra- and intermolecular hydrogen bonds are shown as green dashed lines.

## supplementary crystallographic information

### Comment

Isoindoles and their derivatives are of great pharmaceutical importance (Mancilla *et al.*, 2007). In addition, some derivatives of isoindoles have shown a wide range of herbicidal activities (Toru *et al.*, 1986). Here, the synthesis and characterization with X-ray crystallography of a new derivative is described.

The molecule of the title compound (Fig. 1), is similar to the previously reported compound, 1,3-dimethoxy-2,3-dihydro-1H-isoindole-2-carbothioamide, with its bond lengths and angles being within normal ranges (Maliha *et al.*, 2009). Ring A (C1—C6) is planar, while the five-membered ring B (N1/C5/C6/C7/C8) adopts an envelope conformation with atom N1 displaced by 0.320 (3) Å from the plane of the other ring atoms. The molecule contains a pseudo mirror plane, with the symmetrical orientations of the O-CH_3_ groups leading to R and S-configurations at carbon atoms C7 and C8, respectively. The crystal structure is stabilized by an intramolecular N—H···O interaction and an intermolecular N—H···O interaction (see Table 1), which links a pair of molecules to form a dimer (Fig. 2).

### Experimental

All reagents and solvents were used as obtained commercially without further purification. The title compound was prepared according to the reported procedure (Maliha *et al.*, 2009). For the preparation of the title compound, a mixture of *ortho*-phthaldehyde (1.34 g, 10 mmol) and 2, 4-dinitrophenylhydrazine (1.98 g, 10 mmol) in 20 ml of methanol, and aqueous NaOH (5 ml, 5%) was added dropwise with constant stirring. Then, it was further refluxed in methanol for 2 h, and left to stand overnight. After 3 h, a colorless precipitate was obtained, which was washed with hexane, ethanol and acetone, respectively. Crystals suitable for X-ray analysis were obtained from a solution of acetone/methanol mixture by slow evaporation at room temperature.

### Refinement

H atoms bonded to C atoms were placed geometrically and treated as riding, with C—H distances 0.93–0.98Å and *U*_iso_(H) = 1.2*U*_eq_(C) for the CH while *U*_iso_(H) = 1.5*U*_eq_(C) for the CH_3_ groups. The amide H atoms were located from difference maps and refined with the N—H distances restrained to 0.86 Å and *U*_iso_(H) = 1.2*U*_eq_(N).

### Crystal data

**Table:**

C_16_H_16_N_4_O_6_	*Z* = 2
*M**_r_* = 360.33	*F*(000) = 376
Triclinic, *P*1	*D*_x_ = 1.447 Mg m^−^^3^
Hall symbol: -P 1	Mo *K*α radiation, λ = 0.71073 Å
*a* = 7.727 (4) Å	Cell parameters from 1638 reflections
*b* = 10.244 (5) Å	θ = 2.8–27.5°
*c* = 11.326 (6) Å	µ = 0.11 mm^−^^1^
α = 86.076 (9)°	*T* = 296 K
β = 77.705 (8)°	Block, colorless
γ = 70.794 (8)°	0.16 × 0.14 × 0.10 mm
*V* = 827.2 (7) Å^3^	

### Data collection

**Table:**

Bruker SMART APEXII CCD diffractometer	3181 independent reflections
Radiation source: fine-focus sealed tube	2045 reflections with *I* > 2σ(*I*)
graphite	*R*_int_ = 0.062
phi and ω scans	θ_max_ = 26.0°, θ_min_ = 1.8°
Absorption correction: multi-scan (*SADABS*; Sheldrick, 1996)	*h* = −9→8
*T*_min_ = 0.982, *T*_max_ = 0.989	*k* = −11→12
4365 measured reflections	*l* = −13→13

### Refinement

**Table:**

Refinement on *F*^2^	Primary atom site location: structure-invariant direct methods
Least-squares matrix: full	Secondary atom site location: difference Fourier map
*R*[*F*^2^ > 2σ(*F*^2^)] = 0.067	Hydrogen site location: inferred from neighbouring sites
*wR*(*F*^2^) = 0.225	H-atom parameters constrained
*S* = 1.02	*w* = 1/[σ^2^(*F*_o_^2^) + (0.1489*P*)^2^] where *P* = (*F*_o_^2^ + 2*F*_c_^2^)/3
3181 reflections	(Δ/σ)_max_ < 0.001
237 parameters	Δρ_max_ = 0.49 e Å^−^^3^
0 restraints	Δρ_min_ = −0.25 e Å^−^^3^

### Special details

**Table:**

Geometry. All e.s.d.'s (except the e.s.d. in the dihedral angle between two l.s. planes) are estimated using the full covariance matrix. The cell e.s.d.'s are taken into account individually in the estimation of e.s.d.'s in distances, angles and torsion angles; correlations between e.s.d.'s in cell parameters are only used when they are defined by crystal symmetry. An approximate (isotropic) treatment of cell e.s.d.'s is used for estimating e.s.d.'s involving l.s. planes.
Refinement. Refinement of *F*^2^ against ALL reflections. The weighted *R*-factor *wR* and goodness of fit *S* are based on *F*^2^, conventional *R*-factors *R* are based on *F*, with *F* set to zero for negative *F*^2^. The threshold expression of *F*^2^ > σ(*F*^2^) is used only for calculating *R*-factors(gt) *etc*. and is not relevant to the choice of reflections for refinement. *R*-factors based on *F*^2^ are statistically about twice as large as those based on *F*, and *R*- factors based on ALL data will be even larger.

### Fractional atomic coordinates and isotropic or equivalent isotropic displacement parameters (Å^2^)

**Table:**

	*x*	*y*	*z*	*U*_iso_*/*U*_eq_	
C1	0.1312 (5)	0.1163 (3)	0.6041 (3)	0.0665 (9)	
H1	0.1032	0.0400	0.6417	0.080*	
C2	0.1069 (5)	0.1515 (4)	0.4885 (3)	0.0741 (10)	
H2	0.0623	0.0983	0.4471	0.089*	
C3	0.1476 (5)	0.2646 (4)	0.4328 (3)	0.0684 (9)	
H3	0.1297	0.2869	0.3543	0.082*	
C4	0.2142 (4)	0.3446 (3)	0.4915 (3)	0.0588 (8)	
H4	0.2414	0.4212	0.4539	0.071*	
C5	0.2401 (4)	0.3088 (3)	0.6080 (2)	0.0451 (6)	
C6	0.1985 (4)	0.1974 (3)	0.6640 (2)	0.0487 (7)	
C7	0.2308 (4)	0.1839 (3)	0.7897 (2)	0.0501 (7)	
H7	0.1096	0.2205	0.8450	0.060*	
C8	0.3055 (4)	0.3813 (3)	0.6914 (2)	0.0450 (6)	
H8	0.2013	0.4633	0.7241	0.054*	
C9	0.4404 (3)	0.3127 (2)	0.9664 (2)	0.0404 (6)	
C10	0.4134 (3)	0.3760 (2)	1.0796 (2)	0.0405 (6)	
C11	0.5561 (4)	0.3448 (2)	1.1440 (2)	0.0436 (6)	
H11	0.5367	0.3873	1.2179	0.052*	
C12	0.7242 (4)	0.2512 (3)	1.0975 (2)	0.0478 (7)	
C13	0.7560 (4)	0.1846 (3)	0.9883 (2)	0.0516 (7)	
H13	0.8714	0.1193	0.9589	0.062*	
C14	0.6169 (4)	0.2160 (3)	0.9250 (2)	0.0496 (7)	
H14	0.6395	0.1717	0.8516	0.060*	
C15	0.4939 (5)	−0.0207 (3)	0.7495 (4)	0.0802 (10)	
H15A	0.4677	−0.0681	0.6887	0.120*	
H15B	0.5744	−0.0866	0.7950	0.120*	
H15C	0.5546	0.0439	0.7116	0.120*	
C16	0.6218 (5)	0.3179 (3)	0.5833 (3)	0.0656 (8)	
H16A	0.6744	0.2530	0.6417	0.098*	
H16B	0.7128	0.3590	0.5413	0.098*	
H16C	0.5869	0.2708	0.5265	0.098*	
N1	0.3375 (3)	0.2795 (2)	0.78934 (18)	0.0438 (5)	
N2	0.3060 (3)	0.3424 (2)	0.90125 (19)	0.0499 (6)	
H2A	0.1982	0.4013	0.9289	0.060*	
N3	0.2382 (3)	0.4743 (2)	1.13435 (19)	0.0478 (6)	
N4	0.8726 (4)	0.2154 (3)	1.1652 (3)	0.0671 (8)	
O1	0.1085 (3)	0.5064 (3)	1.0803 (2)	0.0811 (8)	
O2	0.2215 (3)	0.5263 (2)	1.23014 (19)	0.0758 (7)	
O3	1.0248 (4)	0.1387 (4)	1.1204 (3)	0.1139 (12)	
O4	0.8371 (4)	0.2619 (3)	1.2668 (3)	0.1046 (11)	
O5	0.3227 (3)	0.0518 (2)	0.8286 (2)	0.0697 (6)	
O6	0.4614 (3)	0.42259 (18)	0.64289 (17)	0.0553 (6)	

### Atomic displacement parameters (Å^2^)

**Table:**

	*U*^11^	*U*^22^	*U*^33^	*U*^12^	*U*^13^	*U*^23^
C1	0.078 (2)	0.0666 (19)	0.071 (2)	−0.0393 (17)	−0.0207 (17)	−0.0068 (15)
C2	0.078 (2)	0.087 (2)	0.073 (2)	−0.0333 (19)	−0.0310 (18)	−0.0206 (18)
C3	0.077 (2)	0.082 (2)	0.0539 (18)	−0.0243 (18)	−0.0284 (16)	−0.0103 (16)
C4	0.074 (2)	0.0550 (17)	0.0505 (17)	−0.0184 (15)	−0.0220 (14)	−0.0007 (13)
C5	0.0475 (15)	0.0434 (13)	0.0443 (14)	−0.0093 (11)	−0.0157 (11)	−0.0063 (11)
C6	0.0502 (15)	0.0503 (15)	0.0488 (15)	−0.0158 (12)	−0.0156 (12)	−0.0062 (12)
C7	0.0563 (16)	0.0491 (15)	0.0472 (15)	−0.0189 (12)	−0.0116 (12)	−0.0027 (12)
C8	0.0533 (15)	0.0390 (13)	0.0429 (14)	−0.0110 (11)	−0.0147 (11)	−0.0043 (10)
C9	0.0470 (14)	0.0391 (13)	0.0373 (13)	−0.0139 (11)	−0.0139 (11)	0.0029 (10)
C10	0.0463 (14)	0.0361 (12)	0.0380 (13)	−0.0112 (10)	−0.0100 (11)	0.0007 (10)
C11	0.0552 (16)	0.0383 (13)	0.0406 (14)	−0.0156 (11)	−0.0160 (12)	0.0006 (10)
C12	0.0504 (16)	0.0435 (14)	0.0530 (16)	−0.0139 (12)	−0.0208 (12)	0.0029 (11)
C13	0.0493 (16)	0.0480 (15)	0.0534 (16)	−0.0084 (12)	−0.0127 (13)	−0.0026 (12)
C14	0.0523 (16)	0.0522 (15)	0.0425 (14)	−0.0110 (13)	−0.0131 (12)	−0.0060 (12)
C15	0.087 (3)	0.0460 (17)	0.101 (3)	−0.0132 (17)	−0.020 (2)	0.0006 (17)
C16	0.067 (2)	0.0663 (19)	0.0624 (19)	−0.0253 (16)	−0.0027 (15)	−0.0038 (15)
N1	0.0538 (13)	0.0420 (11)	0.0369 (11)	−0.0127 (10)	−0.0142 (9)	−0.0062 (9)
N2	0.0485 (13)	0.0547 (13)	0.0413 (12)	−0.0047 (10)	−0.0136 (10)	−0.0118 (10)
N3	0.0526 (13)	0.0487 (12)	0.0406 (12)	−0.0100 (10)	−0.0152 (10)	−0.0038 (9)
N4	0.0626 (17)	0.0641 (16)	0.0746 (18)	−0.0036 (13)	−0.0367 (14)	−0.0125 (14)
O1	0.0548 (13)	0.1044 (18)	0.0684 (15)	0.0123 (12)	−0.0283 (11)	−0.0361 (13)
O2	0.0744 (15)	0.0890 (16)	0.0526 (13)	0.0012 (12)	−0.0230 (11)	−0.0312 (11)
O3	0.0634 (16)	0.139 (3)	0.122 (2)	0.0166 (17)	−0.0444 (16)	−0.052 (2)
O4	0.0970 (19)	0.111 (2)	0.094 (2)	0.0161 (16)	−0.0634 (16)	−0.0374 (17)
O5	0.0905 (16)	0.0517 (12)	0.0685 (14)	−0.0258 (11)	−0.0194 (12)	0.0151 (10)
O6	0.0670 (13)	0.0474 (11)	0.0574 (12)	−0.0267 (10)	−0.0108 (10)	−0.0039 (9)

### Geometric parameters (Å, °)

**Table:**

C1—C2	1.369 (5)	C10—N3	1.436 (3)
C1—C6	1.390 (4)	C11—C12	1.357 (4)
C1—H1	0.9300	C11—H11	0.9300
C2—C3	1.376 (5)	C12—C13	1.390 (4)
C2—H2	0.9300	C12—N4	1.448 (4)
C3—C4	1.370 (4)	C13—C14	1.358 (4)
C3—H3	0.9300	C13—H13	0.9300
C4—C5	1.384 (4)	C14—H14	0.9300
C4—H4	0.9300	C15—O5	1.430 (4)
C5—C6	1.365 (4)	C15—H15A	0.9600
C5—C8	1.499 (3)	C15—H15B	0.9600
C6—C7	1.486 (4)	C15—H15C	0.9600
C7—O5	1.396 (4)	C16—O6	1.418 (4)
C7—N1	1.473 (3)	C16—H16A	0.9600
C7—H7	0.9800	C16—H16B	0.9600
C8—O6	1.397 (3)	C16—H16C	0.9600
C8—N1	1.472 (3)	N1—N2	1.399 (3)
C8—H8	0.9800	N2—H2A	0.8600
C9—N2	1.343 (3)	N3—O2	1.205 (3)
C9—C14	1.401 (4)	N3—O1	1.227 (3)
C9—C10	1.421 (3)	N4—O3	1.204 (3)
C10—C11	1.388 (4)	N4—O4	1.217 (3)
			
C2—C1—C6	118.4 (3)	C12—C11—H11	120.5
C2—C1—H1	120.8	C10—C11—H11	120.5
C6—C1—H1	120.8	C11—C12—C13	121.7 (2)
C1—C2—C3	121.0 (3)	C11—C12—N4	119.4 (3)
C1—C2—H2	119.5	C13—C12—N4	118.8 (2)
C3—C2—H2	119.5	C14—C13—C12	119.3 (3)
C4—C3—C2	120.8 (3)	C14—C13—H13	120.4
C4—C3—H3	119.6	C12—C13—H13	120.4
C2—C3—H3	119.6	C13—C14—C9	122.3 (2)
C3—C4—C5	118.4 (3)	C13—C14—H14	118.8
C3—C4—H4	120.8	C9—C14—H14	118.8
C5—C4—H4	120.8	O5—C15—H15A	109.5
C6—C5—C4	121.1 (3)	O5—C15—H15B	109.5
C6—C5—C8	110.5 (2)	H15A—C15—H15B	109.5
C4—C5—C8	128.4 (3)	O5—C15—H15C	109.5
C5—C6—C1	120.4 (3)	H15A—C15—H15C	109.5
C5—C6—C7	110.8 (2)	H15B—C15—H15C	109.5
C1—C6—C7	128.8 (3)	O6—C16—H16A	109.5
O5—C7—N1	112.1 (2)	O6—C16—H16B	109.5
O5—C7—C6	117.2 (2)	H16A—C16—H16B	109.5
N1—C7—C6	101.9 (2)	O6—C16—H16C	109.5
O5—C7—H7	108.4	H16A—C16—H16C	109.5
N1—C7—H7	108.4	H16B—C16—H16C	109.5
C6—C7—H7	108.4	N2—N1—C8	112.18 (19)
O6—C8—N1	113.0 (2)	N2—N1—C7	113.7 (2)
O6—C8—C5	117.0 (2)	C8—N1—C7	110.43 (19)
N1—C8—C5	101.6 (2)	C9—N2—N1	121.4 (2)
O6—C8—H8	108.3	C9—N2—H2A	119.3
N1—C8—H8	108.3	N1—N2—H2A	119.3
C5—C8—H8	108.3	O2—N3—O1	121.1 (2)
N2—C9—C14	121.0 (2)	O2—N3—C10	120.2 (2)
N2—C9—C10	122.7 (2)	O1—N3—C10	118.7 (2)
C14—C9—C10	116.3 (2)	O3—N4—O4	122.5 (3)
C11—C10—C9	121.4 (2)	O3—N4—C12	119.1 (3)
C11—C10—N3	116.3 (2)	O4—N4—C12	118.4 (3)
C9—C10—N3	122.3 (2)	C7—O5—C15	114.8 (2)
C12—C11—C10	118.9 (2)	C8—O6—C16	115.8 (2)
			
C6—C1—C2—C3	−0.2 (5)	N4—C12—C13—C14	179.1 (3)
C1—C2—C3—C4	0.2 (5)	C12—C13—C14—C9	−0.6 (4)
C2—C3—C4—C5	0.2 (5)	N2—C9—C14—C13	179.4 (2)
C3—C4—C5—C6	−0.7 (4)	C10—C9—C14—C13	−0.6 (4)
C3—C4—C5—C8	−178.1 (3)	O6—C8—N1—N2	84.6 (3)
C4—C5—C6—C1	0.8 (4)	C5—C8—N1—N2	−149.3 (2)
C8—C5—C6—C1	178.6 (3)	O6—C8—N1—C7	−147.5 (2)
C4—C5—C6—C7	−177.7 (2)	C5—C8—N1—C7	−21.3 (3)
C8—C5—C6—C7	0.2 (3)	O5—C7—N1—N2	−85.2 (3)
C2—C1—C6—C5	−0.3 (5)	C6—C7—N1—N2	148.6 (2)
C2—C1—C6—C7	177.8 (3)	O5—C7—N1—C8	147.7 (2)
C5—C6—C7—O5	−135.8 (3)	C6—C7—N1—C8	21.5 (3)
C1—C6—C7—O5	46.0 (4)	C14—C9—N2—N1	−0.8 (4)
C5—C6—C7—N1	−13.0 (3)	C10—C9—N2—N1	179.2 (2)
C1—C6—C7—N1	168.7 (3)	C8—N1—N2—C9	−122.1 (3)
C6—C5—C8—O6	136.2 (2)	C7—N1—N2—C9	111.7 (3)
C4—C5—C8—O6	−46.1 (4)	C11—C10—N3—O2	1.0 (4)
C6—C5—C8—N1	12.7 (3)	C9—C10—N3—O2	−179.5 (3)
C4—C5—C8—N1	−169.6 (3)	C11—C10—N3—O1	179.2 (2)
N2—C9—C10—C11	−179.0 (2)	C9—C10—N3—O1	−1.4 (4)
C14—C9—C10—C11	1.0 (4)	C11—C12—N4—O3	−175.6 (3)
N2—C9—C10—N3	1.6 (4)	C13—C12—N4—O3	6.7 (5)
C14—C9—C10—N3	−178.4 (2)	C11—C12—N4—O4	6.0 (5)
C9—C10—C11—C12	−0.3 (4)	C13—C12—N4—O4	−171.6 (3)
N3—C10—C11—C12	179.2 (2)	N1—C7—O5—C15	−66.5 (3)
C10—C11—C12—C13	−1.0 (4)	C6—C7—O5—C15	50.8 (3)
C10—C11—C12—N4	−178.6 (2)	N1—C8—O6—C16	64.6 (3)
C11—C12—C13—C14	1.5 (4)	C5—C8—O6—C16	−52.9 (3)

### Hydrogen-bond geometry (Å, °)

**Table:**

*D*—H···*A*	*D*—H	H···*A*	*D*···*A*	*D*—H···*A*
N2—H2A···O1	0.86	1.96	2.593 (3)	130
N2—H2A···O1^i^	0.86	2.27	3.032 (3)	148

Symmetry codes: (i) −*x*, −*y*+1, −*z*+2.
